# The Relationship between Changes in Organ-Tissue Mass and Sleeping Energy Expenditure Following Weight Change in College Sumo Wrestlers

**DOI:** 10.3390/medicina56100536

**Published:** 2020-10-13

**Authors:** Taishi Midorikawa, Shigeho Tanaka, Takafumi Ando, Masayuki Konishi, Megumi Ohta, Suguru Torii, Shizuo Sakamoto

**Affiliations:** 1 College of Health and Welfare, J.F. Oberlin University, 3758 Tokiwamachi, Machida, Tokyo 194-0294, Japan; 2 Waseda Institute for Sport Sciences, Waseda University, 2-579-15 Mikajima, Tokorozawa, Saitama 359-1192, Japan; m-konishi@hm.tokoha-u.ac.jp; 3 Faculty of Nutrition, Kagawa Nutrition University, 3-9-21 Chiyoda, Sakado, Saitama 350-0288, Japan; tanaka.shigeho@eiyo.ac.jp; 4 Human-Centered Mobility Research Center, Information Technology and Human Factors, National Institute of Advanced Industrial Science and Technology, Central 6, 1-1-1 Higashi, Tsukuba, Ibaraki 305-8561, Japan; takafumi.ando@aist.go.jp; 5 Department of Integrated Studies of Human Development and Clinical Psychology, Faculty of Health Promotional Sciences, Tokoha University, 1230 Miyakoda-cho, Kita-ku, Hamamatsu, Shizuoka 431-2102, Japan; 6 School of International Liberal Studies, Chukyo University, 101-2 Yagoto Honmachi, Showa-ku, Nagoya-shi, Aichi 466-8666, Japan; m-ohta@lets.chukyo-u.ac.jp; 7 Faculty of Sport Sciences, Waseda University, 2-579-15 Mikajima, Tokorozawa, Saitama 359-1192, Japan; shunto@waseda.jp (S.T.); s.sakamoto@waseda.jp (S.S.)

**Keywords:** body composition, energy expenditure, longitudinal study, organ-tissue mass, indirect human calorimeter

## Abstract

*Background and objectives*: It has been well established that the resting energy expenditure (REE) for the whole body is the sum of the REE for each organ-tissue in young and middle-aged healthy adults. Based on these previous studies, although it is speculated that sleeping energy expenditure (SEE, which has small inter-individual variability) changes with a commensurate gain or reduction in the resting metabolic rate of each organ-tissue, it is unclear whether a change in organ-tissue masses is directly attributed to the fluctuation of SEE at present. This study aimed to assess the relationship between changes in organ-tissue mass and sleeping energy expenditure (SEE) following weight change in college Sumo wrestlers. This included blood analysis, which is related to energy expenditure. *Materials and Methods*: A total of 16 healthy male college Sumo wrestlers were recruited in this study. All measurements were obtained before and after weight change. Magnetic resonance imaging measurements were used to determine the volume of the skeletal muscle (SM), liver, and kidneys, and an indirect human calorimeter was used to determine SEE before and after weight change. *Results*: The change in body mass and SEE ranged between −8.7~9.5 kg, and −602~388 kcal/day. Moreover, changes in SM, liver, and kidneys ranged between −3.3~3.6 kg, −0.90~0.77 kg, and −0.12~0.07 kg. The change in SEE was not significantly correlated with the change in SM or liver mass, nor with blood analyses; however, a significant relationship between the change in kidney mass and SEE was observed. *Conclusions*: Based on our results, there is a possibility that the mass of the kidneys has an effect on the change in SEE following weight change in college Sumo wrestlers.

## 1. Introduction

It has been well established that the resting energy expenditure (REE) for the whole body is the sum of the REE for each organ-tissue in young and middle-aged healthy adults [1,2,3]. Moreover, in the case of male college Sumo wrestlers with high REEs (i.e., about 2300 kcal/day), larger absolute amounts of low and high metabolically active tissue, including the skeletal muscle (SM), liver, and kidneys, are responsible for REE [4]. In addition, our previous study demonstrated that aerobic endurance training does not lead to a chronic elevation in each organ-tissue’s resting metabolic rate (kcal/kg/day) in cases with a relatively high $\overset{\cdot }{V}$O_2_ peak (i.e., approximately 60 mL/min/kg) [5]. Therefore, since the resting metabolic rate of each organ-tissue might remain unchanged following moderate exercise training, the increase or decrease of organ-tissue mass would be a key factor for changing REE.

According to previous studies, it has been revealed that the mass of internal organs such as the liver and kidneys changed as a result of diet and exercise. A recent study involving diet and exercise weight-loss intervention found decreases in the mass of the SM, liver, and kidneys (approximately 1.0 kg, 0.1 kg, and 0.01 kg, respectively) after a 6.2 kg weight reduction [6]. Moreover, in a longitudinal study of collegiate male American football players, the mass of the liver and kidneys increased by 0.2 kg and 0.04 kg, respectively, after 1 year of overfeeding and physical training [7]. Based on these previous studies, although it is speculated that REE changes with a commensurate gain or reduction in the resting metabolic rate of each organ-tissue (e.g., 200 kcal/kg/day for the liver; 440 kcal/kg/day for kidneys) [8], it is unclear whether changes in internal organs can be directly attributed to the fluctuation of REE at present.

Therefore, we aimed to assess the relationship between the change in organ-tissue mass and sleeping energy expenditure (SEE) following weight change in college Sumo wrestlers. Although Sumo wrestlers are specific subjects, our previous cross-sectional study found that they had higher REE values of the SM, liver, and kidneys than controls [4], and we would expect to observe a larger fluctuation of the SM and internal organs. At present, though it is generally accepted that a change in REE and SEE is mainly induced by a gain or loss in fat-free mass, it is still unclear whether these changes are due to changes in SM mass, internal organs of fat-free mass components, or other parameters. This study included blood analysis, which is related to energy expenditure. Although SEE is generally slightly lower than REE in the lying position, the inter-individual variability in SEE is reported to be small, probably due to the accuracy of an indirect human calorimeter (IHC) for measurements and can be considered “the stable condition” [9]. An understanding of the relationship between body composition and SEE following weight change may yield valuable information about energy requirements.

## 2. Materials and Methods

### 2.1. Subjects

A total of 16 healthy male college Sumo wrestlers aged 18–22 years were recruited in this study. All measurements were obtained before and after weight change (mean length of time between measurements: 31 ± 12 months). College Sumo wrestlers had performed regular training (“Kei-ko”) for more than 10 years. Kei-ko normally consists of wrestling exercises and additional technical drills that include a mix of power, agility, and endurance training [10]. None of the subjects had a history of cardiovascular, endocrine, or orthopedic disorders, nor had been taking any medication related to body composition or energy expenditure. All the subjects received a verbal and written description of the study and provided informed consent before testing. The study protocol was approved by the Ethical Committee of Waseda University (project identification code: 2012–113; date of approval: 21 August 2012) and the National Institute of Health and Nutrition (project identification code: 20130201-01; date of approval: 1 February 2013) and was conducted according to the guidelines laid down in the Declaration of Helsinki.

### 2.2. Anthropometry and Body Composition Measurements Using Dual-Energy X-ray Absorptiometry and Magnetic Resonance Imaging

Total body mass, fat mass, and lean soft tissue mass (LSTM) were measured using DXA (Delphi A-QDR, Hologic Inc., Bedford, MA, USA; Version 12.4:3 Auto Whole Body Fan Beam). The estimated coefficient of variation (CV) was <1%, based on the intra-observer variability for assessing fat mass and LSTM, employing DXA from the test–retest analyses conducted previously. Height was measured on a stadiometer (YS-OA, AS ONE Co. Ltd.) to the nearest 0.1 cm. Body mass index (BMI; kg/m^2^) was calculated as body mass (kg) using DXA divided by the square of the height (m).

The volume of whole-body SM and the internal organs (liver and kidneys) were estimated using a General Electric Signa EXCITE VI 1.5 Tesla scanner (Milwaukee, WI, USA). A T1-weighted spin-echo, axial-plane sequence was employed, with a 500 ms repetition time and a 13.1 ms echo time, during breath-holding scans and normal breathing scans. Subjects rested quietly in the magnet bore in the supine position with their hands placed on their abdomen. Contiguous transverse images with 1.0 cm slice thicknesses (0 cm interslice gap) were obtained from the top of the head to the malleolus lateralis for each subject. Approximately 5 acquisition sets were performed, extending from the top of the head to the femoral head, as subjects held their breath (approximately 30 s/set). A second set of acquisitions were obtained from the femoral head to the malleolus lateralis during normal breathing [11]. All images (approximately 155 slices per person) were traced by a highly trained technician, including the SM and abdominal organ segments and excluding connective tissue, blood vessels, and fat tissue. Magnetic resonance images were analyzed using ZedView software (LEXI Co., Ltd., Tokyo, Japan) for segmentation and calculation of the cross-sectional tissue areas. Since the large body frame produced artifacts in the arm region, the SM mass for arms from the axillary fossa to the end of the finger was excluded from the analysis.

The volume of the SM, liver, and kidneys was calculated from the sum of the cross-sectional area (cm^2^), determined by tracing the images and then multiplying the value by the slice thickness (1 cm). The volumes (cm^3^) were converted to masses (kg) using the following densities: 1.041 g/cm^3^ for the SM [12], 1.060 g/cm^3^ for liver, and 1.050 g/cm^3^ for kidneys [13]. The estimated CV for SM volume measurements from a test–retest analysis was 2% [11]. The percentage of difference in measurements for the same scan on 2 separate days by the same technician was 0.3% for the liver and 0.5% for the kidneys (*n* = 5). Anthropometry and body composition were obtained during a single-study visit to Waseda University.

### 2.3. Blood Collection and Analysis

Blood samples were collected in tubes containing thrombin for analyzing the levels of thyroid-stimulating hormone (TSH), total and free T_3_, and total and free T_4_, as well as into tubes containing ethylenediaminetetraacetic acid disodium salt (EDTA-2Na) for analyzing the levels of plasma epinephrine and norepinephrine. The blood samples in both tubes were centrifuged (3000 rpm for 10 min) after collection, and the serum and plasma were transferred into plastic tubes. Serum and plasma samples were immediately stored at < −30 °C for further analysis. The concentrations of serum TSH and total and free T_3_ and T_4_ were measured using an electrochemiluminescence immunoassay (accuracies are <18% for TSH, <15% for total T_3_ and T_4_, and <20% for free T_3_ and T_4_, and CVs for all assays are <10%). Plasma epinephrine and norepinephrine concentrations were measured using high-performance liquid chromatography. Blood samples were analyzed by SRL, Inc. (Tokyo, Japan).

### 2.4. Sleeping Energy Expenditure (SEE) Measurements Using Indirect Human Calorimeter (IHC)

The subjects entered the IHC chamber at 18:00 on the study day, had dinner at 18:30, went to bed at 22:00 after sedentary activities, and slept until 06:00 the following morning [9]. Each subject was provided with a standardized dinner (1283 kcal, Protein: Fat: Carbohydrate balance = 15.4:23.4:61.1) to meet their energy expenditure (EE) during their stay in the chamber. None of the subjects performed any exercise during the 48 h prior to testing. Details of the IHC method have previously been published [14,15,16]. The values of $\overset{\cdot }{V}$O_2_ and $\overset{\cdot }{V}$CO_2_ were recorded under conditions of standard temperature and pressure and dry conditions. Energy expenditure was estimated from $\overset{\cdot }{V}$O_2_ and $\overset{\cdot }{V}$CO_2_ using Weir’s equation [17]. The accuracy and precision of the IHC for measuring EE, as determined by the alcohol combustion test, was 99.8% ± 0.5% (mean ± standard deviation (SD)) in 6 h and 99.4% ± 3.1% in 30 min. SEE was defined as the minimum EE recorded over 3 h of sleep between 23:00 and 07:00.

### 2.5. Statistical Analysis

Results are expressed as mean ± SD for all variables. The difference in subject characteristics, organ-tissue mass, and blood analyses between before-and-after weight change were analyzed using a paired *t*-test. Spearman rank correlation analyses were used to describe the relationships between changes in SEE and MRI-measured SM mass and internal organs, and blood analyses. Stepwise multiple regression analysis was also performed to analyze change in SEE and these factors. Statistical analyses were performed using SPSS for Windows (IBM SPSS version 25.0; SPSS Inc., Chicago, IL, USA). A *p*-value of <0.05 was considered to be statistically significant.

## 3. Results

**Figure 1:**
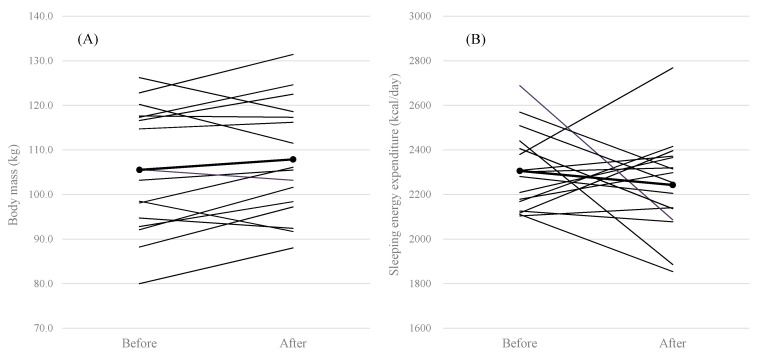

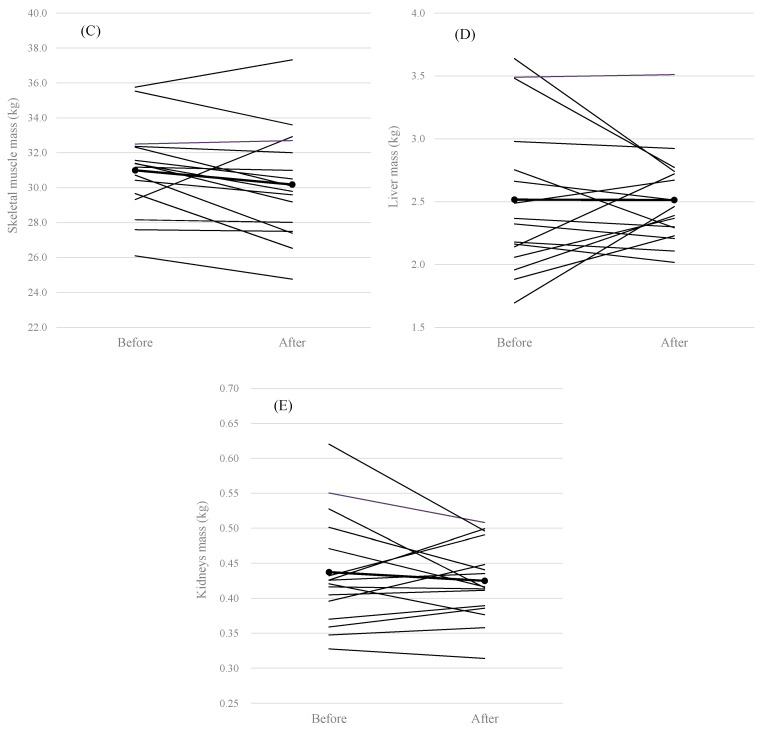
The changes in body mass (**A**), sleeping energy expenditure (**B**), skeletal muscle mass except for arm regions (**C**), liver mass (**D**), and kidneys mass (**E**) following weight change.

**Figure 2:**
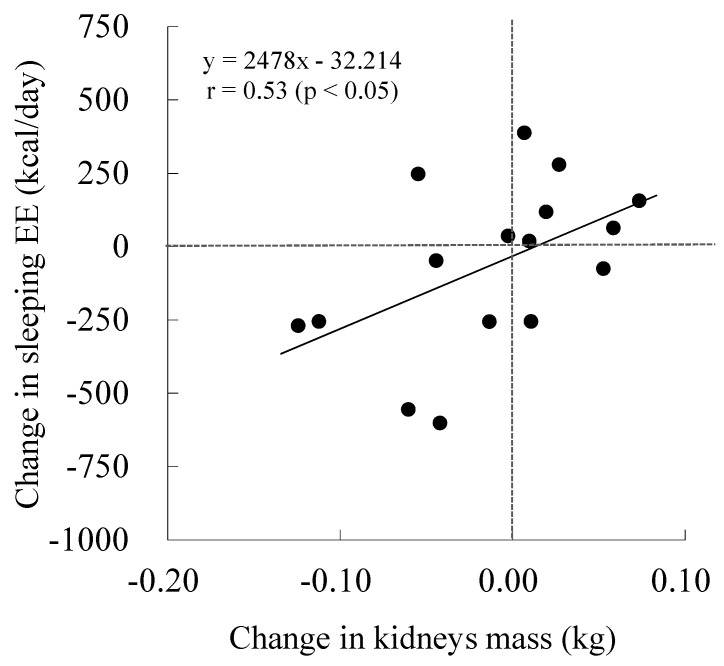
The relationship between changes in kidney mass and sleeping energy expenditure (EE).

There were no significant differences in subject characteristics and mass of SM, liver, or kidneys and blood analyses between before-and-after weight change, except for standing height (Table 1, Table 2 and Table 3). The change in body mass and SEE ranged between −8.7~9.5 kg and −602~388 kcal/day (Figure 1). Moreover, changes in the SM, liver, and kidneys ranged between −3.3~3.6 kg, −0.90~0.77 kg and −0.12~0.07 kg (Figure 1). The change in SEE was not significantly correlated with the change in SM or liver mass, nor with blood analyses; however, a significant relationship between the change in kidney mass and SEE was observed in all subjects (Table 4, Figure 2). In addition, stepwise regression analyses indicated that the change in kidney mass was independently associated with that in SEE.

## 4. Discussion

To our knowledge, this is the first longitudinal study regarding the relationship between changes in organ-tissue mass and SEE. Based on our results, there is a possibility that kidney-mass has an effect on the change in SEE following weight change. According to a limited study regarding the longitudinal effects of organ-tissue mass on REE, a gain or loss in kidney mass additionally explained the variance of REE change in stepwise multiple regression analysis [18]. Although the change in kidney mass is insignificant (between −0.04 and +0.02 kg in the current study) compared to other factors, it is probable that the influence of the kidneys, coupled with a high resting metabolic rate (i.e., 440 kcal/kg/day in Reference Man), on the variation of SEE is significant in cases of weight change. Moreover, there were two cases of increasing SEE despite decreasing kidney mass, and vice versa in this study. While these cases do not mean that there is causality between the changes in kidney mass and SEE, it is a point worthy of investigating in further studies with larger populations and more variation in kidney mass.

As is the case with the kidneys, the liver has high metabolic activity (i.e., 200 kcal/kg/day). However, a significant relationship between the change in liver mass and SEE was not indicated in the present study. Similarly, a relationship between the change in liver mass and REE following weight change was not found in previous longitudinal studies [18,19]. Moreover, according to a previous study that measured liver fat content using single-voxel proton magnetic resonance spectroscopy (^1^H-MRS), liver fat content was found to have a high variance, between 0.4% and 30.4% [19]. Based on these results, as liver fat content could be a confounding factor, we would need to accurately measure hepatic cells alone, to determine if there is an effect on REE and/or SEE following weight change.

It has long been thought that fluctuation of fat-free mass resulted in a change in SM mass and that internal organs such as the liver and kidneys remained unchanged following weight change. Moreover, previous studies reported that exercise-training induced changes in fat-free mass were significantly correlated with variations in REE following strength training [20,21]. Based on these findings, it has generally been accepted that a gain or loss in SM mass induces a change in REE and/or SEE following total body resistance training. However, it is still unclear whether exercise training-induced changes in REE were directly caused by changes in SM mass (i.e., 13 kcal/kg/day). Although the present study has attempted to answer this question, the effect of changes in SM mass on the variation of SEE was not observed. On the other hand, the limited previous study revealed that changes in SM mass were a key factor affecting fluctuating REE during changes in weight without exercise training [18]. Based on the findings of the previous study, although it is speculated that changes in SM mass are related to REE variation following exercise training, further study is needed to fully determine the relationship.

In the present study, we observe that the range of changing SEE was relatively large (i.e., approximately 400 kcal/day) following weight gain and loss. The variation in SEE was larger than the value predicted from changing organ-tissue mass and resting metabolic rate (e.g., 13 kcal/kg/day for SM; 440 kcal/kg/day for kidney). Although previous studies have suggested that changes in T_3_ correlated with changes in REE [18], the hormonal change was not clearly observed in the present study. Moreover, according to our previous studies, children aged 6–12 have a higher organ-tissue resting metabolic rate (kcal/kg/day) than adults [22]. On the other hand, cross-sectional studies have reported that the absolute value of REE and/or SEE would be attributed to the organ-tissue mass and that the metabolic rate is constant among athletes, including college Sumo wrestlers aged 19 [4,5]. Based on these findings, we concluded that college Sumo wrestlers in the present study are not in that growth phase. Further studies are required to ascertain whether each organ-tissue metabolic rate fluctuates following weight change by using noninvasive methods such as positron emission tomography.

## 5. Conclusions

The goal of this study was to determine whether changes in SEE following weight change were due to changes in organ-tissue mass. Based on our results, there is a possibility that the mass of kidneys has an effect on the change in SEE following weight change in college Sumo wrestlers. Since the results of this longitudinal study are not fully in accordance with previous cross-sectional studies and are based on moderate correlations in a small sample size (*n* = 16) and specific subjects, future clinical studies are needed with generic subjects and larger sample sizes to investigate hypertrophy of the SM and internal organs following weight change. Although we could not conclusively answer these questions, we hope that the results we obtained pave the way for others to understand the relationships between organ-tissue mass and REE and/or SEE.

## Figures and Tables

**Table 1:** Subject characteristics and sleeping energy expenditure following weight change.

	Pre			Post			Change		
Age (year)	19	±	1	22	±	1	3	±	1
Standing height (cm)	172.4	±	4.9	173.0	±	4.7	0.5	±	0.7 *
Body mass (kg)	108.4	±	14.4	109.7	±	12.6	1.3	±	6.9
BMI (kg/m^2^)	36.4	±	3.9	36.6	±	3.7	0.2	±	2.1
Fat (%)	25.0	±	4.7	26.1	±	4.3	1.1	±	3.7
Fat mass (kg)	27.7	±	8.3	29.0	±	7.9	1.3	±	5.4
Lean soft tissue mass (kg)	77.5	±	6.9	77.3	±	6.4	−0.1	±	3.7
SEE (kcal/day)	2306	±	179	2243	±	220	−63	±	285

BMI: body mass index; SEE: sleeping energy expenditure. Pre vs. Post: * *p* < 0.05.

**Table 2:** Organ-tissue mass following weight change.

Organ-Tissue Mass (kg)	Pre			Post			Change		
Skeletal muscle ^1^	31.00	±	2.56	30.18	±	3.10	-0.82	±	1.74
Liver	2.52	±	0.60	2.51	±	0.37	0.00	±	0.44
Kidneys	0.44	±	0.08	0.42	±	0.05	-0.01	±	0.06

^1^: Whole body skeletal muscel mass except for arm regions. Pre vs. Post: n.s. for each organ-tissues.

**Table 3:** Blood analyses following weight change.

	Pre			Post			Change		
TSH (μIU/mL)	3.0	±	2.0	3.7	±	3.0	0.7	±	3.2
Total T3 (ng/mL)	1.2	±	0.1	1.3	±	0.3	0.1	±	0.3
Free T3 (pg/mL)	3.7	±	0.3	3.8	±	0.9	0.1	±	1.0
Total T4 (μg/dL)	7.6	±	1.0	7.6	±	1.8	0.1	±	1.8
Free T4 (ng/dL)	1.3	±	0.2	1.5	±	0.3	0.2	±	0.4
Epinephrin (pg/mL)	50.7	±	23.3	42.4	±	23.4	−8.3	±	28.2
Norepinephrin (pg/mL)	432.5	±	173.3	477.5	±	235.6	45.0	±	223.9

TSH: thyroid stimulation hormone, T3: triiodothyronine, T4: throxine. Pre vs. Post: n.s. for each blood analyses.

**Table 4:** Correlation coefficients between change in SEE and organ-tissue & blood analyses.

	SM	Liver	Kidneys	TSH	Total T3	Free T3	Total T4	Free T4	Epinephrin	NE
Liver	0.42									
Kidneys	0.32	0.07								
TSH	0.17	−0.18	−0.14							
Total T3	−0.15	−0.16	0.04	−0.26						
Free T3	−0.24	0.04	0.00	−0.17	0.87 **					
Total T4	−0.33	−0.35	−0.42	−0.23	0.73 **	0.62 *				
Free T4	−0.28	0.03	−0.39	−0.37	0.72 **	0.83 **	0.79 **			
Epinephrin	−0.54 *	−0.49	−0.16	0.17	0.21	0.32	0.46	0.25		
NE	−0.36	0.03	−0.18	0.32	0.19	0.41	0.34	0.31	0.43	
SEE	0.28	0.38	0.53 *	−0.05	0.19	0.21	−0.07	−0.14	0.11	0.18

TSH: thyroid stimulation hormone, T3: triiodothyronine, T4: throxine, NE: norepinephrin, SEE: sleeping energy expenditure. * *p* < 0.05, ** *p* < 0.01.
